# Molecular Targets for Antibody-Based Anti-Biofilm Therapy in Infective Endocarditis

**DOI:** 10.3390/polym14153198

**Published:** 2022-08-05

**Authors:** Jiahe Han, Alessandro Poma

**Affiliations:** 1 UCL Institute of Cardiovascular Science, The Rayne Building, 5 University Street, London WC1E 6JF, UK; 2 Division of Biomaterials and Tissue Engineering, UCL Eastman Dental Institute, Royal Free Hospital, UCL Medical School, Rowland Hill Street, London NW3 2PF, UK

**Keywords:** infective endocarditis, biofilms, antibodies, immunotherapy, epitopes, antibiotic resistance

## Abstract

Infective endocarditis (IE) is a heart disease caused by the infection of heart valves, majorly caused by *Staphilococcus aureus*. IE is initiated by bacteria entering the blood circulation in favouring conditions (e.g., during invasive procedures). So far, the conventional antimicrobial strategies based on the usage of antibiotics remain the major intervention for treating IE. Nevertheless, the therapeutic efficacy of antibiotics in IE is limited not only by the bacterial drug resistance, but also by the formation of biofilms, which resist the penetration of antibiotics into bacterial cells. To overcome these drawbacks, the development of anti-biofilm treatments that can expose bacteria and make them more susceptible to the action of antibiotics, therefore resulting in reduced antimicrobial resistance, is urgently required. A series of anti-biofilm strategies have been developed, and this review will focus in particular on the development of anti-biofilm antibodies. Based on the results previously reported in the literature, several potential anti-biofilm targets are discussed, such as bacterial adhesins, biofilm matrix and bacterial toxins, covering their antigenic properties (with the identification of potential promising epitopes), functional mechanisms, as well as the antibodies already developed against these targets and, where feasible, their clinical translation.

## 1. Introduction

### 1.1. The Background of IE and Its Modern Epidemiology

Infective endocarditis (IE) is an infectious heart disease, with an incidence of three to ten episodes per 100,000 per year in the population [1,2,3]. Over the years, the mortality of IE changed with advances in medicine: IE was indeed a highly fatal disease up to the 1940s, when the introduction of penicillin greatly reduced its mortality [4,5]. Later on, the development of valvular surgery sparked new modes of intervention, defining the beginning of the early surgery era [6]. Although the majority of IE cases can be treated with antibiotics, about 25–30% of patients develop severe valvular damage or exhibit ineffectiveness in response to antibiotic treatments, and therefore surgery is required during the early-acute phase of infection. Another 20 to 40% of these patients require surgery later [7].

Concurrently, the ongoing development of antibiotics and surgical interventions renews our understanding of the epidemiology of IE. The changes in antibiotics have altered the patterns of infection and caused resistance worldwide [8,9,10]. On the other hand, in comparison with the pre- and early antibiotic age in which rheumatic heart disease was a leading cause of IE, nowadays, other risk factors are taken into consideration, such as the use of intracardiac devices, prosthetic valve replacement, venous catheters, or haemodialysis. Moreover, the average age of IE patients and the prevalence of comorbidities among them have increased [11,12,13].

At the time of writing, IE is still a serious disease for its high mortality, since 20% of patients die in hospital, and its one-year mortality rate is about 30% [1,2]. Despite the development of trends toward earlier diagnosis and early surgery, the mortality of IE has barely improved in the last four decades, and its one-year mortality has not improved in over two decades [7,14]. So far, the current management of IE is still challenged by difficulties in both prognosis and treatment.

### 1.2. The Clinical Pathology of IE

**Figure 1:**
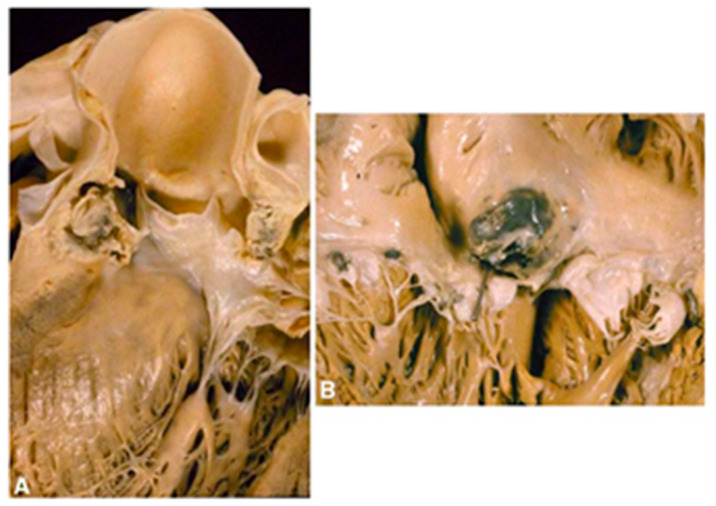
The local spreading of IE infection. IE originates from the aortic valve and can spread to the ventricular wall (**A**) and atrium wall (**B**). Adapted with permission from Thiene and Basso [16]. 2006, Elsevier.

As an infectious heart disease, IE is initiated by the bacterial infection of heart valves. Bacteria can enter the bloodstream *via* the skin, the mouth, gastrointestinal and urinary tracts, through venous catheters or after an invasive medical or surgical procedure. After that, bacteria can reach the heart *via* body circulation and then adhere to the valves. This will lead to the formation of holes on the valve, or scarring of the valve tissue, which consequently will (or can) result in valve leakage [7,15]. The common signs and symptoms of IE include: chest pain, fatigue, flu-like symptoms and abnormal heart murmurs [15]. The characteristic lesions of IE are vegetations consisting of microorganisms, inflammatory cells, platelets, fibrin and leaflet disruptions. IE might be extended by local spread of the infection, which leads to other complications (Figure 1) [16].

Since IE can occur on both original and prosthetic valves, it is further classified as native valve endocarditis (NVE) and prosthetic valve endocarditis (PVE). In the case of NVE, deformed or damaged endothelium is more accessible for bacterial infection than the healthy one, and IE could develop following injury or thrombosis. Indeed, non-bacterial thrombotic endocarditis (NBTE), which is distinguished from IE for its non-infective pathogenesis, has now been identified as a crucial factor underlying the development of IE. Sterile thrombotic vegetations caused by NBTE are predisposed for bacterial adhesion on valve surfaces; therefore, the entry of microorganisms into the mainstream circulation could convert NBTE into IE. Further, injury to the endocardium and vascular endothelium may generate predilection sites of infection even in the absence of sterile thrombotic vegetations [17].

PVE is initiated by microorganisms invading the prosthesis during surgery or following haematogenic dissemination after surgery (a rare and serious complication of valve replacement). Similar to the pathology of NVE, thrombosis also acts as an underlying inducement for PVE. Thrombus formation on the prosthetic valve counts on a range of factors, including endothelisation, haemodynamics and haemostasis [18]. First of all, the endothelisation of a prosthetic valve takes several weeks to be completed. Therefore, in the early post-operative period, the absence of endothelium could raise the thrombotic risk. Secondly, the altered haemodynamics after valve replacement can contribute to generating turbulent flow in localised regions, which can then lead to stasis and thrombosis on the prosthetic valve. Additionally, the clotting on the valve caused by blood stasis is critical to thrombus formation, and therefore anticoagulant treatment is required for patients after surgery. Eventually, the thrombus composed of fibrin and thrombocytes can serve as a predisposing focus of infection. Taking all of these factors into consideration, although NVE is distinguished from PVE, there are a number of similarities in their pathologies.

### 1.3. The Challenges in Antibiotic Treatment and Prospective Anti-Biofilm Strategies

IE can be caused by a range of micro-organisms, but *Staphylococci* are now considered the most frequent causative organisms, since they represent the major pathogens for hospital-acquired infections [4,15,19,20,21,22,23]. Therefore, *Staphylococci* have become the main targets for antibiotic therapy for this condition. However, the therapeutic efficiency of antibiotics for IE has been challenged by the emergence of resistant *Staphylococci* since their early introduction. As an example, only a few years after the introduction of penicillin, resistant staphylococcal strains expressing β-lactamase were reported [24]. The introduction of methicillin (a synthetic β-lactamase-resistant penicillin) facilitated the development of Methicillin-resistant *Staphylococcus aureus* (MRSA) strains [25,26,27]. To compete with the insurgence of resistant bacterial strains, new antibiotics and different combinations of synergistic antibiotics have been tested to improve IE treatment. Importantly, in addition to the acquired drug resistance, virulence factors also critically contribute to the modulation of antibiotic susceptibility in *Staphylococci*; indeed, IE is particularly difficult to treat due to the ability of MRSA to hinder the action of antibiotics *via* the formation of biofilms [28,29].

**Figure 2:**
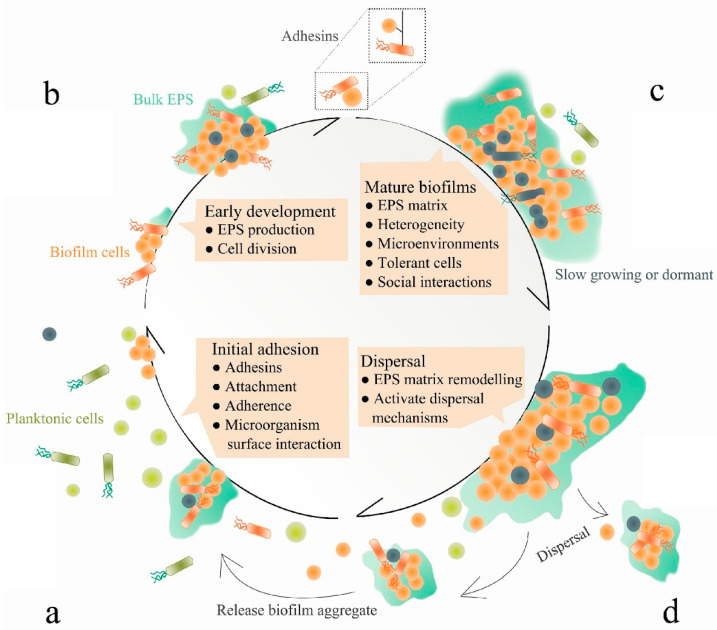
The molecular mechanisms of biofilm formation. Attachment (**a**), biofilm formation (**b**), maturation (**c**) and dispersion (**d**). Adapted from Jiang et al. [34]. The different colours and shapes model the different bacterial cells composing the biofilm.

A biofilm can be described as a sessile community of micro-organisms in which cells embed together by attaching to others and a surface in a protective extracellular polymeric matrix, also known as extracellular polymeric substance (EPS). The extracellular matrix of biofilms can effectively increase tolerance to antibiotics *via* multiple mechanisms [30]. The EPS in staphylococcal biofilms is composed of polysaccharides, extracellular DNA (eDNA) and/or proteins [17,18,31,32,33]. The formation of a biofilm can be generally summarised into three main stages: attachment, maturation and dispersion (Figure 2).

Firstly, attachment is initiated once an individual planktonic bacterial cell reversibly associates with a surface, and such association will become irreversible if the cell remains in contact for a sufficient period of time. Bacterial attachment is mediated by surface proteins such as microbial surface components recognising adhesive matrix molecules (MSCRAMMs) [35]. However, the importance and involvement of these proteins varies largely between strains, and many of these factors function in both attachment and accumulation [34]. Following attachment, biofilm maturation is progressed by bacterial division and their production of the extracellular polymeric matrix, enabling the transition of bacteria from a planktonic to a sessile state. Finally, bacteria within the biofilm can return to the planktonic state; hence, they can disassemble to undergo dispersion [36].

All stages of biofilm formation can be targeted for anti-biofilm therapeutic purposes. For example, the initial attachment can be prevented by targeting staphylococcal adhesions [37]; biofilm maturation can be disturbed by blocking surface proteins involved in cell-to-cell adhesion [38]; and finally, the pre-existing biofilm can be decomposed by using dispersal agents, such as *cis*-2-decenoic acid (C2DA), dispersin B and DNase I [35].

Since anti-biofilm strategies target the molecular pathways involved in the biofilm’s formation and maturation, which is different from the conventional antimicrobial routes, this translates into a significantly reduced selection pressure, which in turn mitigates the potential development of resistance. Further, since factors involved in staphylococcal biofilm formation are highly species-specific (compared to targets for conventional antibiotics), anti-biofilm strategies may allow for the development of narrow-spectrum precision agents, which will have low or no influence on other microbiota [34]. To date, a wide range of molecular targets involved in biofilm formation are being investigated, and the combination of antibiotics and anti-biofilm therapies is likely to be more effective than a single treatment.

### 1.4. Antibodies as a Promising Approach for Anti-Biofilm IE Treatment

**Figure 3:**
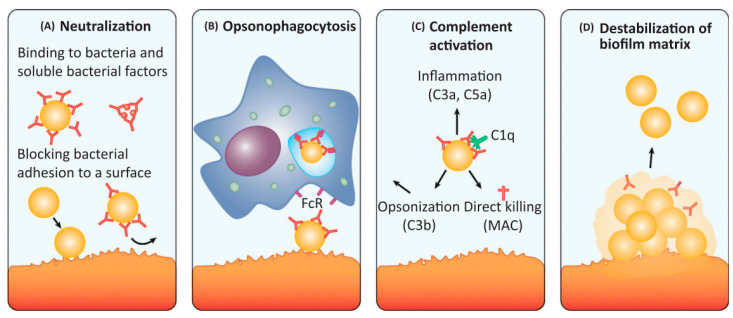
The antibodies against biofilm can disrupt ongoing biofilm formation and/or disperse established biofilms by different mechanisms: (**A**) neutralisation, (**B**) opsonophagocytosis, (**C**) complement activation and (**D**) destabilisation of biofilm matrix. Adapted with permission from Raafat et al. [42]. 2019, Elsevier.

In addition to the molecular agents that could inhibit staphylococcal biofilm formation, different alternative strategies can be used, such as monoclonal antibodies (mAbs) treatment. When staphylococcal infections occur, the immune system generates antibodies against a wide range of antigens, including surface proteins, toxins and cell wall proteins [36,39,40,41]. These antibodies can disrupt the biofilm’s formation *via* different immunological mechanisms (Figure 3).

A neutralising antibody is an antibody that can block the infectious and pathogenic effects of microbes. High-affinity IgA and IgG antibodies can neutralise the action of secreted staphylococcal proteins, such as immune evasion molecules, exoenzymes, toxins and surface proteins, which are potential therapeutic targets. Further, neutralising antibodies can in turn bind to bacterial adhesins (e.g., MSCRAMMs) and cell wall components to inhibit initial attachment to host matrixes and subsequent initiation of biofilm formation (Figure 3A). Another type of mechanism, known as opsonophagocytosis (i.e., opsonophagocytic-killing, OPK), entails antibodies that can mediate microbial clearances by phagocytes. These surface-bound antibodies (mainly IgG) can trigger OPK by neutrophils and macrophages expressing FcR on their surface. Once antibodies bind to the antigens, their Fc regions are able to activate phagocytes to engulf pathogens (Figure 3B). Similarly, microbial clearance can also be mediated *via* activating the complement system. The classical complement pathway is activated once the surface-bound antibodies (IgM and IgG) bind to the C1q subunit on the C1 complex, and then the following cascade leads to the formation of the C3 convertase, which cleaves C3 (the central component of all complement pathways) into C3a and C3b. Finally, C3b acts as an opsonin to enable phagocytosis *via* C3b receptor-expressed phagocytes ingesting C3b-coated pathogens; the soluble C3a (also known as C5a) acts as chemoattractant to recruit immune cells to initiate inflammation. C3 activation also causes the formation of the membrane attack complex (MAC) that can mediate lysis of certain pathogens. These all contribute to the killing of pathogens (Figure 3C). Additionally, antibodies targeting different components of the biofilm matrix can directly destabilise the biofilm structure and thus promote bacterial dispersal (Figure 3D).

The antibodies naturally produced by the host are highly specific, therefore highlighting the potential of antibody therapy as a feasible option for narrow-spectrum anti-biofilm treatment. So far, the feasibility of antibody therapies disrupting staphylococcal biofilms has been shown by some studies, in which different antigens have been targeted, such as surface proteins [43,44,45,46,47], toxins [45], cell wall enzymes [48] and poly-*N*-acetyl-β-(1,6)-glucosamine (PNAG) [47]. Importantly, the major disadvantage of antibodies therapy, as in the case of antibiotics, is related to the poor penetration into the deepest layers of a biofilm, which would result in incomplete disruption. Moreover, development of resistance is possible, although less likely than with antibiotics. Another hurdle faced by the development of antibodies for anti-biofilm IE treatment is that different *Staphylococci* strains express different antigens; therefore, finding a universal target is difficult, which renders the antibody development quite challenging [49].

This review, therefore, aims to identify the most promising targets for anti-biofilm IE treatment, with particular emphasis on antibody-based therapy. We will do so by summarising and evaluating the potential targets for anti-biofilm IE treatment, highlighting the most promising ones for antibody development and discussing the challenges in antibody design and development for IE treatment.

## 2. Molecular Targets for Monoclonal Antibodies Targeting *Staphylococcus* Biofilms

**Figure 4:**
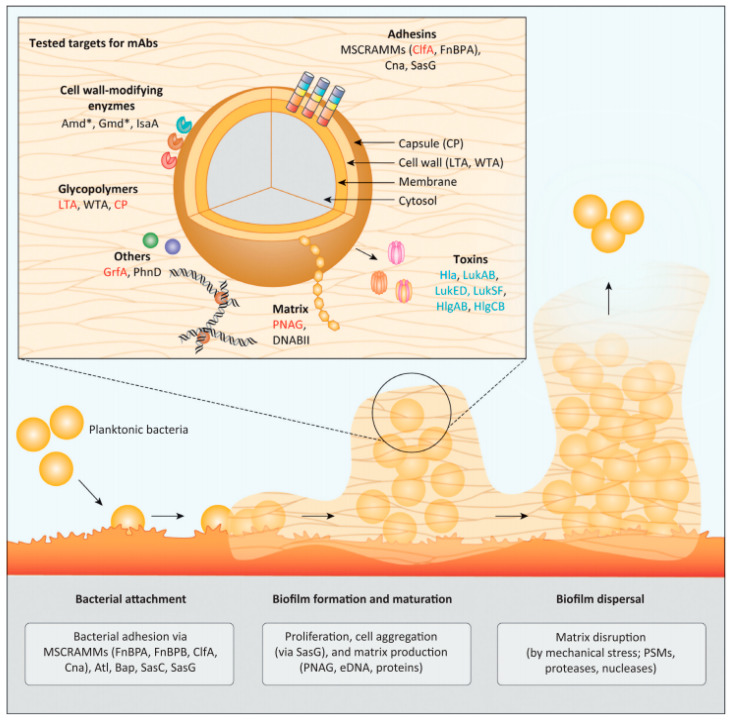
Potential molecular targets involved in *Staphylococcus* biofilm formation. The biofilm could be disrupted by generating antibodies against functional proteins involved in each stage of biofilm formation. Adapted with permission from Raafat et al. [42]. 2019, Elsevier.

So far, the natural immunological reactions triggered by biofilm-associated infection are not well understood. Importantly, host antibodies stimulated by *S. aureus* antigens show reduced efficiency in preventing a reinfection with this pathogen [50]. As mentioned above, the process of biofilm formation can be summarised into three stages in which a wide range of functional proteins are involved. Given the importance of biofilm formation in the pathology of IE, current research on antibodies targeting *S. aureus* infections has included anti-biofilm strategies. So far, some bacterial proteins contributing to *S. aureus* biofilm formation have been considered as candidates for developing IE treatments, such as adhesins, biofilm matrix components, cell wall-modifying enzymes, surface glycopolymers and toxins (Figure 4).

The functions of these candidates as well as their clinical studies are summarised in Table 1.

## 3. Anti-Biofilm Strategies

### 3.1. Inhibition of Bacterial Attachment

The first stage of biofilm formation, bacterial attachment, relies on a number of staphylococcal surface-binding proteins, including MSCRAMMs and other cell wall-associated proteins [e.g., biofilm-associated protein (Bap), *S. aureus* surface proteins C (SasC) and G (SasG)] (Figure 4) [92]. The initial attachment *in vivo* is mainly driven by the interaction between MSCRAMMs and the human extracellular matrix (Figure 4) [93]. Therefore, MSCRAMMs are taken as candidates for IE antibody-based therapies and, ideally, their antibodies are supposed to function *via* a mechanism that prevents the initial bacterial adherence to both abiotic and biotic surfaces by neutralising adhesins and/or promoting OPK [50,84,94].

Among the family of MSCRAMMs, ClfA, ClfB and the fibronectin-binding proteins (FnBPA and FnBPB), are widely found among the *S. aureus* strains, and other proteins such as collagen-binding protein (Cna) only distribute in a subset of strains [95]. Importantly, however, in comparison to MSCRAMM ClfA, ClfB presents a relatively high frequency of pseudogenes (i.e., DNA sequences that resemble a gene but have been mutated into an inactive form over the course of evolution). This reveals its decreasing importance in the MSCRAMMs family, therefore indicating that ClfB is less suitable as a target for IE antibodies [96].

#### 3.1.1. ClfA: Past Failure of Anti-ClfA Antibodies Enlightens Further Research

The MSCRAMM ClfA is a virulence factor that critically contributes to the colonisation of *S. aureus* on protein-coated biomaterials and damaged endothelial surfaces by binding to blood plasma protein fibrinogen (Fg). Further, ClfA is found to predominantly promote staphylococcal adhesion under high shear stress [97]. To understand the properties of ClfA that render it an effective target for IE, it is critical to consider its functional mechanism and protein structure, as well as its genetic variations.

**Figure 5:**
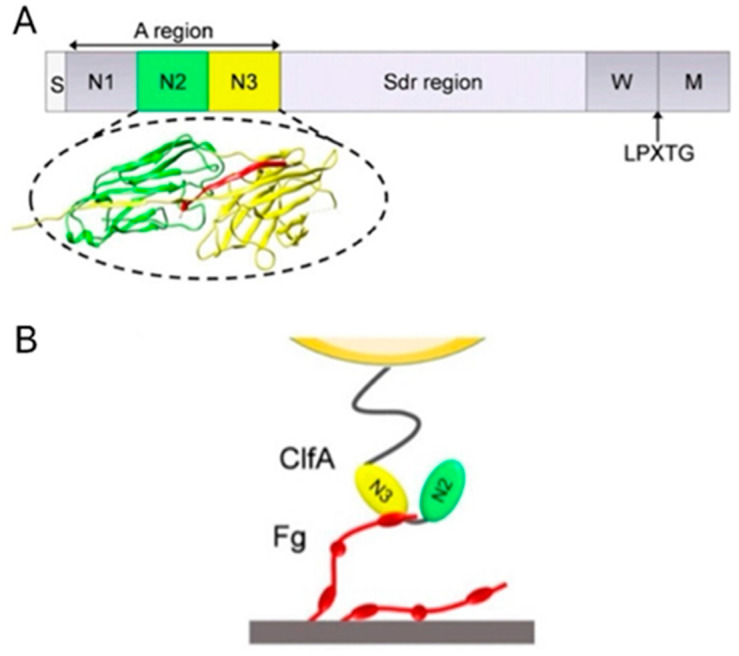
The protein structure of ClfA and its functional mechanism. (**A**) From the *N-* to *C-* terminus, ClfA is composed of signal sequence (S), A region with subdomains N1, N2 and N3, repeated serine-aspartate residues (Sdr) region, wall spanning (W) region, LPXTG motif (LPXTG) and sorting sequence (M). The binding site of Fg is between N2 (green) and N3 (yellow). (**B**) The γ-chain of Fg (red) binds to the trench between N2 and N3. Adapted from Herman-Bausier et al. [97].

As a member of MSCRAMMs, ClfA shares a similar domain organisation and structure. Starting from the *N*-terminus, ClfA contains a signal sequence followed by a ligand-binding *N*-terminal A region (amino acids 40 to 559) subdivided into independently folded N1, N2 and N3 subdomains, a region consisting of repeated serine-aspartate residues, and a C-terminal region containing an LPXTG motif (Figure 5) [98,99,100].

These components are responsible for its structural function and mechanism. The junction between N2 and N3 is found as a binding trench where the ligand inserts and binds with ClfA. The Sdr region links region A to the C-terminal wall-spanning region and the sorting sequence. The LPXTG motif allows anchoring of the protein to cell wall peptidoglycan by sortase A. The ClfA—Fg interaction occurs *via* ClfA binding its minimal ligand-binding segment N2 and N3 to the carboxy-terminus of the γ-chain of Fg through variations of a dynamic mechanism termed “Dock, Lock and Latch” (DLL): Firstly, the carboxy-terminus of the γ-chain of Fg docks in a ligand-binding trench located between subdomains N2 and N3 (amino acids 221 to 559). Once the ligand peptide docks into the trench, it is subsequently covered by a flexible C-terminal extension of the N3 domain and thus “locked” in place [101]. After that, the C-terminal part of this extension interacts with the N2 domain and forms an extra β-strand, which complements the pre-existing β-sheet in the N2 domain, and together they serve as a latch to stabilise the MSCRAMM—ligand complex.

The diversity of ClfA has been explored by studies on its variants. The results obtained from structural mapping of CflA subdomains reveal a minimum pairwise identity of 86%, which indicates a low level of structural differences among variants. Therefore, ClfA is considered to be well-conserved, and also one single isolate is predicted to be able to generate cross-reactive antibody responses against a wide range of variants [96]. However, this predication is questioned by a further study, which has shown that ClfA strains present lower binding affinities to heterologous antibodies elicited by their variants, with only 10% variation in aminoacid sequences [102]. This result highlights that small differences in composition across *S. aureus* strains could result in large effects on antigenicity.

Interestingly, as a member of the MSCRAMM family, *S. aureus* ClfA shows force sensitivity, and its binding to Fg is significantly enhanced by mechanical force, which shows that the ClfA—Fg binding is increased 15-fold in the presence of mechanical tension [97]. According to the same study, ClfA interacts with Fg *via* two distinct binding sites, and the stronger binding site is favoured by high shear stress [97]. This mechanical sensitivity of ClfA shows high biological significance to PVE. As mentioned in Section 1.2, the pathology of PVE is critically related to the changed haemodynamics after aortic valve replacement, which causes turbulent flow that can lead to elevated shear stress levels in the ascending aorta [103]. The elevated shear stress caused by the implantation of the prosthetic valve has been proved by a series of past studies, and a maximal stress of 500 N/m^2^ has been indicated [103,104]. This can probably benefit *S. aureus* attachment on the prosthetic valve through enhancement in ClfA—Fg binding. However, the confirmation of a potential association between the mechanical sensitivity of ClfA and the pathology of PVE would require deeper investigations.

For the reasons mentioned above, antibody treatments targeting MSCRAMM ClfA could possibly prevent the incidence of IE and especially PVE, and indeed, the therapeutic potential of anti-ClfA antibodies has been evidenced by many studies [44,50,51,84,105]. The application of tefibazumab, a humanised anti-ClfA mAb, contributed to the prevention of IE in a rabbit model [44]. However, tefibazumab has failed to achieve statistically significant improvement in a phase II human clinical trial (ClinicalTrials.gov Identifier: NCT00198289). Surprisingly, the tefibazumab epitope is shown to be located on top of the N3 domain instead of the trench discussed above [106]. Strikingly, this is consistent with the dual mechanisms model of ClfA under mechanical tension. Under low tensile force, Fg binds to the top of the ClfA N3 domain *via* weak bonds, whereas under high mechanical tension, extension and conformational changes in the ClfA molecule trigger the ultra-strong DLL interaction by the N2 and N3 subdomains, and the γ-chain peptide of Fg inhibits high forces but not low forces binding site model [97]. Therefore, it can be hypothesised that tefibazumab inhibits the function of ClfA by disrupting its low tension-dependent mechanism instead of the DLL mechanism that responds to a high shear stress environment. This hypothesis, to some extent, could also explain the failure of the phase II clinical trial, as *S. aureus* is exposed to many different levels of shear depending on its location and the type of infectious disease in patients. Notably, although tefibazumab shows therapeutic efficiency in an IE rabbit model, the medical scenario in patients might be much more complex due vascular ageing, calcification and the accompaniment of other cardiovascular diseases [107]. Studies also show that tefibazumab inhibits binding of Fg to ClfA rather than the Fg γ-chain, and residues P467A, Y512A and W518A in the N3 domain are shown to be critically involved in this binding. This provides additional target sites for the future design of effective inhibitors of the ClfA/Fg interaction. Further, as mentioned above, the failure of tefibazumab could be partially explained by its weakened binding affinity when confronting CflA variants from different strains of *S. aureus* [102,106]. A new anti-ClfA mAb (11h10) was identified by the group of Tkaczyk et al., and its combination with anti-toxin mAb shows more improved efficacy than single-neutralising mAb in responding to *S. aureus* biofilm infection [50,84]. The same research group also reported another anti-ClfA mAb (SAR114) with >100-fold increased affinity, as well as its combined construction with anti-toxin mAb, in the form of a bispecific antibody (BisAb) [45]. However, to the best of our knowledge, these two anti-ClfA mAbs have not been further investigated since their last publication in 2017. Importantly, though, functional antibodies generated using recombinant ClfA antigens can sufficiently alter the ligand-binding activity of ClfA [108].

#### 3.1.2. FnBP: The Possibility of Developing FnBP Antibody Is Waiting to Be Addressed

**Figure 6:**
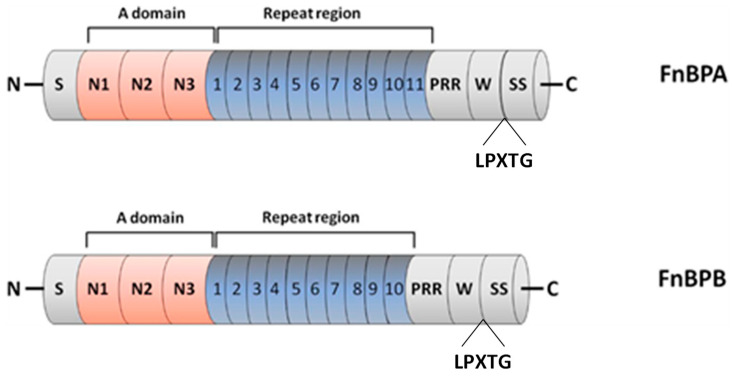
Domain organisation of FnBPA and FnBPB. From the N- to C- terminus, the FnBPs are composed of signal sequence (S), A domain with subdomains N1, N2 and N3, repeat region that is an unstructured region consisting of tandemly arranged motifs (11 in FnBPA and 10 in FnBPB), proline repeated region (PRR), wall-spanning (W) region, LPXTG motif (LPXTG) and sorting sequence (SS). Adapted with permission from O’Neill et al. [54]. 2008, American Society for Microbiology.

FnBP plays multivalent roles in biofilm-associated *S. aureus* infection, as it not only contributes to bacterial adhesion by binding to human plasma proteins, but also promotes bacterial invasion, intercellular accumulation and biofilm maturation [54,93]. In accordance with MSCRAMM ClfA, FnBP shares a similar protein composition, and it also follows the DLL mechanism mediated by the A domain binding site when interacting with Fg (Figure 6).

FnBPA and FnBPB proteins also contain a signal sequence at the N-terminus and an A domain composed of three separately folded subdomains, termed N1, N2 and N3, as well as a wall-spanning region and a sorting signal at the C-terminus. Interestingly, an extra binding site of Fg on the top of the N3 subdomain is also identified in FnBP [106]. In contrast to ClfA, it contains a binding site of fibronectin (Fn) in the repeat region, and it has another binding site of plasminogen (Plg) in the A domain, as a single subdomain was required for Plg binding to FnBPs: subdomain N2 for FnBPA and subdomain N3 for FnBPB [109]. Additionally, it exhibits a much greater level of diversity in its subdomains in comparison to ClfA [110,111]. Noticeably, FnBP can promote biofilm formation, but the underlying multivalent mechanisms are still unclear. This has been firstly explained by a low affinity binding between the A domains of FnBP on adjacent bacteria, which can contribute to the aggregation of bacteria [54]. However, a recent study shows that FnBP can contribute to biofilm formation through a previously unknown mechanism that is distinct from its ligand-binding ability as a member of the MSCRAMMs family [112]. Considering its multivalent functions, FnBP is suggested to be a potent candidate to prevent *S. aureus* infection.

Early studies have highlighted the potential of FnBP antibody therapies, as well as its OPK activity [108,113]. Later, the efficacy of FnBP antibodies was investigated *in vivo*. An anti-FnBPB mAb (15E11) has been identified in a murine model, with a successful inhibition of bacterial attachment by 70% [114]. The mAb 15E11 binds to an epitope shared by the repeated regions in both FnBPA and FnBPB, which is proximal to (though distinct from) the Fn binding site. Furthermore, the sequence KYEQ(H)GGNIV(I)D in the epitope is thought to be crucial. The authors suggested that the steric hindrance or a conformational change elicited by 15E11 reduces the accessibility of Fn to its binding site. This also provides an extra pathway of interference with the action of FnBP, although further studies are required to clarify its underlying mechanism. In addition, an extra binding site of the Fg ligand on the top of the N3 subdomain is also observed in FnBP, and a force-induced binding mechanism similar to that of ClfA has been suggested [115]. So far, though, no FnBP antibodies are being investigated in clinical trials.

### 3.2. Decomposition of Biofilm Matrix

After successful attachment to the surface, bacteria start to proliferate and build biofilm by producing a series of EPSs, including polysaccharides (e.g., PNAG), nucleic acids [e.g., environmental DNA (eDNA)], proteins, lipids and other biomolecules (Figure 4). These matrix proteins support the structural integrity of the biofilm by developing a three-dimensional architecture, which in turn enhances biofilm tolerance to both antimicrobial agents and immune cells. So far, two EPSs, PNAG and DNABII, have been extensively considered as potential candidates for IE antibody-based treatment against biofilm-associated infections. They are currently evaluated as candidates for broad-spectrum antimicrobial therapeutics. This is partly due to the critical roles they play in biofilm composition, as well as their wide distributions and conserved properties observed among a variety of microbes, including bacteria, fungi and protozoa [33,113,116,117].

#### 3.2.1. PNAG: The Antibody against PNAG/dPNAG Shows Optimal Anti-Biofilm Effect

**Figure 7:**
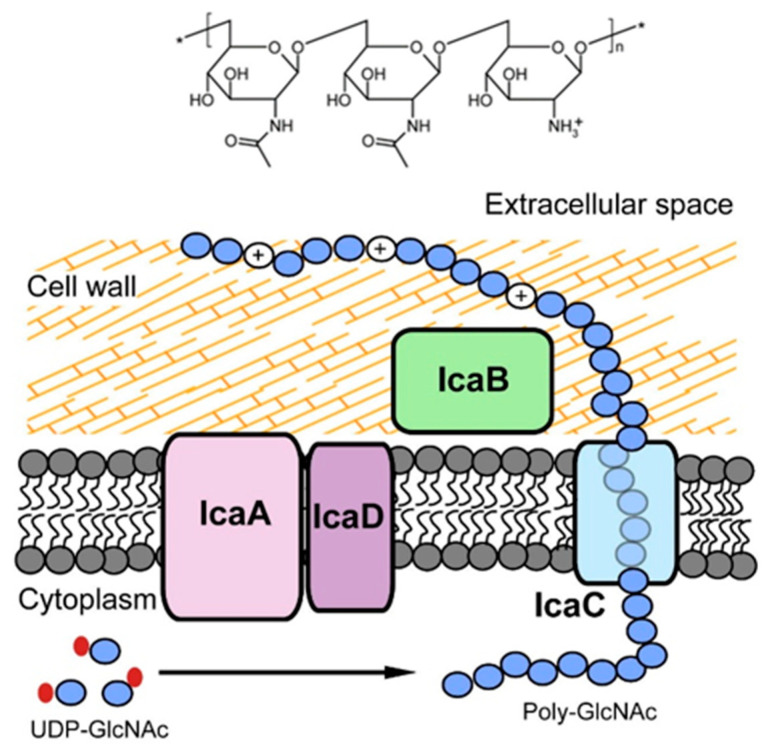
Chemical structure of PNAG (top) and the Ica system (bottom), which is responsible for the synthesis, translocation and deacetylation of PNAG. Adapted with permission from Nguyen et al. [119]. 2020, Elsevier.

PNAG is a major component of the biofilm EPS, also known as polysaccharide intercellular adhesin. PNAG critically mediates intercellular adhesion, thereby leading to the accumulation of bacterial cells, which eventually promote the establishment of biofilms. PNAG not only contributes to the biofilm matrix architecture on the implanted material surface, but also slows down the host defensive responses [118]. The chemical structure of PNAG is shown in Figure 7.

In the case of Gram-positive bacteria, the PNAG synthesis is mediated by the icaADBC locus consisting of four genes, which express four proteins assembling the intercellular adhesion system (Ica) including IcaA, IcaB, IcaC and IcaD (Figure 7). IcaA and IcaD exert primary roles in the exopolysaccharide synthesis. IcaA is a transmembrane enzyme with *N*-acetylglucosaminyl transferase activity, necessary for the synthesis of the PNAG polymer. However, the enzymatic activity of the product of the icaA gene becomes significant, and oligomers longer than 20 residues are synthesised only when co-expressed with the product of the icaD gene. IcaC translocates the PNAG polymer to the bacterial cell surface, and IcaB operates the deacetylation of the molecule.

Deacetylation of PNAG enables its fixation to the outer bacterial surface, promoting the structural development of exopolysaccharide-based biofilm. Importantly, although PNAG is certainly a critical element of biofilm formation in *S. aureus*, the existence of PNAG-independent biofilms has also been confirmed. Furthermore, it has been shown that a minor proportion of *S. aureus* strains can form biofilms even in the absence of the ica locus, which further suggests the existence of ica-independent PNAG-synthesis pathways [120]. As a carbohydrate antigen, the epitope of PNAG is expected to encompass parts of more than one repeating unit, and is often located at the ends of polysaccharide chains [121].

The feasibility of targeting PNAG has been illustrated by many studies [113,122]. Further, different from antibodies against PNAG that aim to decompose biofilm, the antibodies against dPNAG (deacetylated poly-N-β-(1-6)-acetyl-glucosamine) present marked efficacy in the opsonisation and killing of *S. aureus* [81,123,124]. Thus, dPNAG is so far considered a significant target for the development of antibodies aimed at treating IE. However, PNAGs naturally expressed on bacterial surfaces are highly acetylated (>90%) [125]. Structural studies of PNAG indicate that the acetates are on the polymer’s surface and sticking outwards into environment, exposing themselves as the primarily accessible antigens and thus enabling the immunodominance of the acetate-dependent epitopes [126].

As further shown by recent studies, the conjugation of anti-PNAG and anti-dPNAG antibodies achieving significant OPK and protective effects *in vitro* and *in vivo* shows the potential of combined antibodies therapy [117,127]. A human IgG1 mAb (F598, formerly SAR 279356) targeting both PNAG and dPNAG is undergoing preclinical and clinical assessments as a broad-spectrum antimicrobial therapy, since it triggers superior OPK and protective activities against a wide range of microbial pathogens compared to two mAbs that bound optimally to PNAG and minimally to dPNAG (mAbs F628 and F630) [79]. This phenomenon has been explained on the basis of the mAbs’ conformational significance. Once the two Fc regions from two antigen-bound antibodies bind to a C1 complex, the classical pathway of complement cascade is initiated, which is the main killing pathway for many Gram-positive bacterial species (Figure 3C). In the case of PNAG antibodies, as mentioned before, due to the outward positions of their acetate epitopes, the distances between the two Fc regions might not be adequate to bind to a C1 complex and thus fail to activate the complement cascade. However, the binding between the Fc regions of antibodies against both PNAG and dPNAG to the C1 complex is less restricted since their epitopes are located more randomly [126].

**Figure 8:**
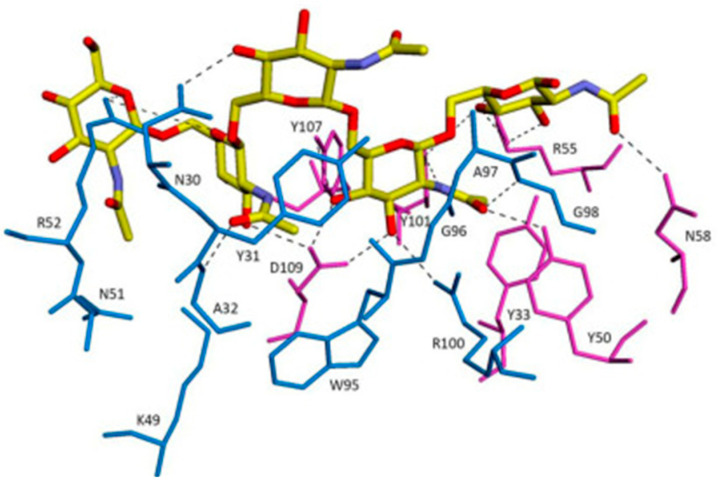
The overall interactions between F598 antigen-binding fragment (Fab)–GlcNAc and Fab–9NAc crystal structures. Fabs are depicted as thin sticks for the L (blue) and H (magenta) chains. Carbohydrate ligands are shown as thicker sticks with carbon atoms of GlcNAc in green and 9NAc in yellow. Adapted with permission from Soliman et al. [128]. 2014, IUCr.

F598 recognises PNAG through a large groove-shaped binding site accommodating five *N*-acetyl-D-glucosamine (GlcNAc) residues as a penta-saccharide epitope, and their interaction is stabilised by two hydrogen bonds linking Asp-109H of F598 to the O3 and O4 atoms of the core GlcNAc (Figure 8).

The Fab arms can span at least 40 GlcNAc residues on an extended PNAG chain [128]. Unfortunately, the clinical trial of F598/SAR 279356 was terminated due to difficulty in patient recruitment (ClinicalTrials.gov Identifier: NCT01389700). Recently, though, the advantage of the anti-biofilm strategy mentioned above has been proved by two studies showing that antibodies against PNAG do not perturb host microbial diversity [129,130]. The current research trends are aimed to develop antibodies against PNAG towards bacterial clearance instead of neutralisation, and both acetate-dependent and -independent epitopes are involved. This, however, brings difficulty to the antibody production process, since the ideal products are proposed to bind both the antigen and C1 complex.

#### 3.2.2. DNABII: A Promising Antibody Target for Anti-Biofilm Treatment

Similar to PNAG, eDNA is also widely distributed among various microbes and critically involved in the construction of biofilm matrix as a part of EPS. The eDNA potentially acts as an electrostatic polymer that anchors cells to a surface *via* the negative charge it carries [129]. The structural integrity of those eDNAs in the biofilm matrix is conversely supported by bacterial DNA-binding proteins (DNABII family). The DNABII family includes HU proteins and integration host factor (IHF), condensing bacterial DNA and also acting as regulators in many cellular processes. HU and IHF have conserved homologs in a wide variety of bacterial species, and they share structural features and the key activity of DNA-bending [131].

The members of the HU family are typically small bacterial proteins (16~20 KDa) and exist as homodimers in Gram-positive bacteria such as *S. aureus*. The *S. aureus* HU (SHU) consists of a hydrophobic core composed of two α-helices and two negatively charged β-sheets arms, and therefore can be divided into two portions: the α-helical “body” and the β-ribbon “arms”. HU acts similarly to a histone through binding and supercoiling DNA into a circular structure; in addition, it also critically acts as a molecular glue, packing eDNA and stabilising the bacterial biofilm by similar patterns [132,133]. This glue-like ability for eDNA is supported by functional characteristics of HU, including a non-specific DNA binding profile [134], high abundance [135] and its ability to migrate into the extracellular medium through multiple mechanisms [124,136,137]. Another DNABII member, IHF, is also known as a DNA-“bending” protein that can create kinks in DNA strands. The DNA-binding events of IHF are more sequence-specific and are only observed in Gram-negative bacteria [138]. Overall, HU can be considered a potential target to decompose *S. aureus* biofilms, and the study of its interactions with DNA can assist the development of anti-DNABII antibodies.

**Figure 9:**
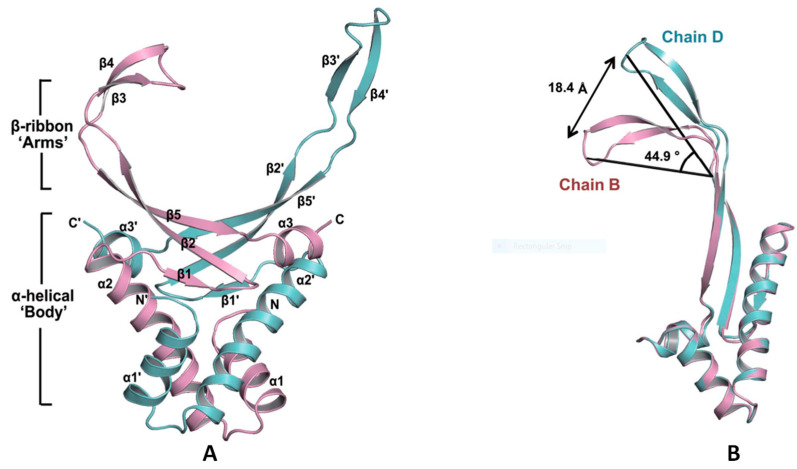

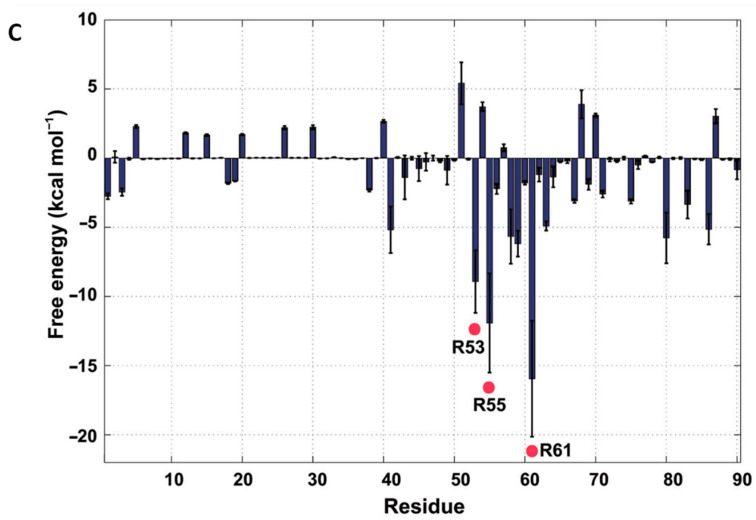
HU structure and the arginine residues involved in its DNA-binding site. (**A**) Ribbon diagram of the SHU homodimer. The α–helices, β–sheets and the N– and C–termini are labelled. The residue Arg55 is labelled in a circle. (**B**) The flexible β–ribbon arms deviate with a distance of 18.4 Å and with a bending angle of approximately 44.9°. (**C**) The binding free energies in the SHU–DNA complex, as determined by MD simulation. The three Arg residues (Arg53, Arg55 and Arg61) that show low free energies are marked with red points. Adapted from Kim et al. [139].

The structure and the DNA-binding mechanisms of SHU have been established (PDB ID: 4QJU). The high flexibility of β-arm is observed in the binding of DNA, and the residue Arg55 positioned in the hinge region of the β-arm exhibits a critical role in their flexible nature. The C atom of Arg55 from chain D is used as the vertex of the angle (Figure 9A,B) [139].

Further, as shown by molecular dynamics analysis on the SHU—DNA complex, the β-arms residues Arg53, Arg55 and Arg61 present low free energy, especially for the Arg61, which suggests their essential role in recognising and binding DNA (Figure 9C). Based on these results, it can be summarised that these three arginine residues on the β-arms are essential for β-arm flexibility, which affects the DNA binding as well as the biological function. Additionally, the involvement of the arginine residues on the β-arms is universal in HU homologues. Interestingly, though, the two essential residues, Arg55 and Arg61, are not completely conserved, whereas Arg58 is conserved in all HU homologues even though it exhibits a relatively small affinity contribution to DNA binding. Further understanding the mechanism of these essential residues might provide us with helpful indications for the design of anti-biofilm agents including but not limited to anti-DNABII antibodies.

Based on its abundance and promising eDNA binding function among different bacteria, DNABII is considered a potent candidate for the development of antibodies. Neutralising of DNABII by specific antibodies has been shown to decompose the biofilm and thus promote the clearance of bacteria by antibiotics and immune cells [140]. Further, targeting DNABII attains high therapeutic efficiency in a wide range of biofilm-associated infections *in vivo*, which significantly indicates its therapeutic potential for polymicrobial IE in a clinical scenario [123,130,132,140]. So far, a native human mAb (TRL1068) generated by Estellés et al. shows anti-biofilm efficacy in two biofilm-associated infectious models that are in the setting of medical devices invasion [81,85]. The epitope of TRL1068 is part of the β-sheet capped with a β-turn in HU, and a specific binding sequence of AARKGRNPQTGKEID within the DNA-binding domain of HU has been identified [81]. This is consistent with the functional importance of β-arm mentioned before. Currently, TRL1068 is undergoing clinical trials (ClinicalTrials.gov Identifier: NCT04763759).

### 3.3. Targeting S. aureus Toxins as Supplemental Therapy

The formation of biofilms is partly assisted by various bacterial toxins, such as pore-forming toxins (e.g., α-toxin (AT), Leukocidin A/B (LukAB) and γ-hemolysin (HlgAB and HlgCB)), and some even produced higher amounts than that by planktonic cells (Figure 4) [85]. Since the pore-forming toxins could counter anti-biofilm immune response *via* mediating lysis of host immune cells, it has been postulated that mAbs neutralising toxins might promote host defences and clearance of planktonic and biofilm cells. AT is the most extensively studied target among these bacterial toxins.

#### AT Antibody: The Only Type of Antibody Currently Successful in Clinical Trials

**Figure 10:**
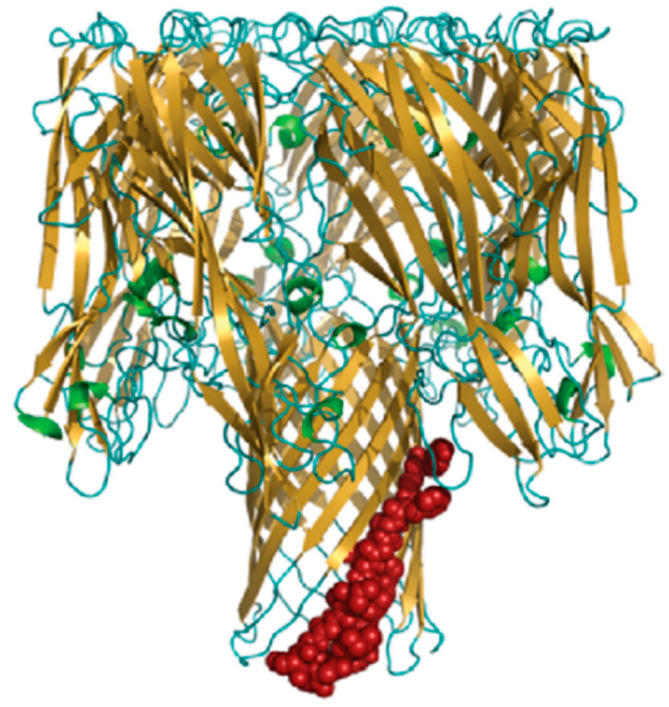
The structure of AT heptameric complex. Adapted from Oscherwitz et al. [141].

*S. aureus* AT is a molecule of ~33 kDa, which exerts its virulence upon a two-step-mediated mechanism. It first binds to its receptor (ADAM10) on the surface of host immune cells and endothelial cells. After that, AT molecules undergo a conformational change to promote oligomerisation, which in turn results in the formation of membrane pores, followed by cell lysis and tissue damage. The AT heptameric complex is comprised of two cylinders: a wider cylinder that comprises the cap and rim domains, and a narrow cylinder called the “pore-forming region” (Figure 10).

The N-terminal 20 amino acids in the cap region of seven monomers interact with each other to lock in a heptameric conformation, and each monomer contributes two β-strands to assemble a fourteen-stranded anti-parallel β-barrel that forms a pore in the cell membrane. The rim domains appear to be proximal to the membrane, as they are directly involved in AT binding to cell [86]. The important residues involved in its pore-forming mechanism have been identified, including N-terminal Arg66 and Glu70 and C-terminal Arg200, Asp254, Asp255 and Asp276 [142]. Furthermore, a determinative sequence of AT pore forming is identified, which could be referred to as a linear neutralising epitope: it is located in the β-barrel pore-forming region of AT, likely including residues 122 to 137 [141].

In addition to lysing immune and host cells, AT can contribute to biofilm formation by promoting bacterial survival, destroying the host epithelium and facilitating bacterial cell-to-cell interactions. Therefore, the antibody against AT is suggested to be a multi-mechanistic anti-biofilm strategy. This suggestion is strongly supported by studies on human mAb (MEDI4893) [85]. This study shows MEDI4893 could exert its neutralising effect through a dual mechanism, as it not only blocks the binding between AT and its cellular receptor ADAM10, but it also inhibits its heptameric conformational change that enables cell lysis [85]. Furthermore, MEDI4893 has been extensively tested in a series of biofilm-associated infection models [84,85]. So far, a phase II clinical trial of MEDI4893 has been completed, which confirms its efficacy in preventing *S. aureus* infection (ClinicalTrials.gov Identifier: NCT02296320). In addition, another AT-neutralising antibody named as AR-301 has successfully passed its phase IIa clinical trial on patients with hospital-acquired bacterial pneumonia (ClinicalTrials.gov Identifier: NCT01589185). Overall, AT antibodies show promising effects on reducing *S. aureus* virulence, which can be used in conjunction with anti-biofilm therapy for achieving higher therapeutic effects.

**Figure 11:**
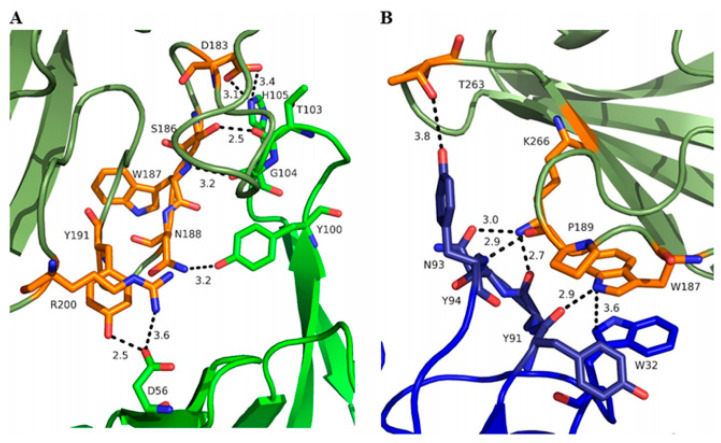
Interface between MEDI4893 HC (green) and AT (olive) (**A**) and MEDI4893 LC (blue) and AT (olive) (**B**). Both HC and LC are in close contact with the rim of AT and create several hydrogen bonds (dotted lines) and one stacking interaction between MEDI4893 Fab Trp32 (LC) and AT Trp187. AT residues in contact with MEDI4893 are shown in orange. Adapted from Oganesyan et al. [86].

The molecular interaction between AT and MEDI4893 has been studied by Oganesyan et al. [86]. MEDI4893 binds to a novel epitope that is a rim domain of AT, comprised of residues Asn177 to Arg200 and Thr261 to Lys271, and the residues within these regions were further confirmed as both functionally and structurally important for MEDI4893 binding. The attachment between the rim and AT is mediated by several hydrogen bonds (Figure 11).

**Figure 12:**
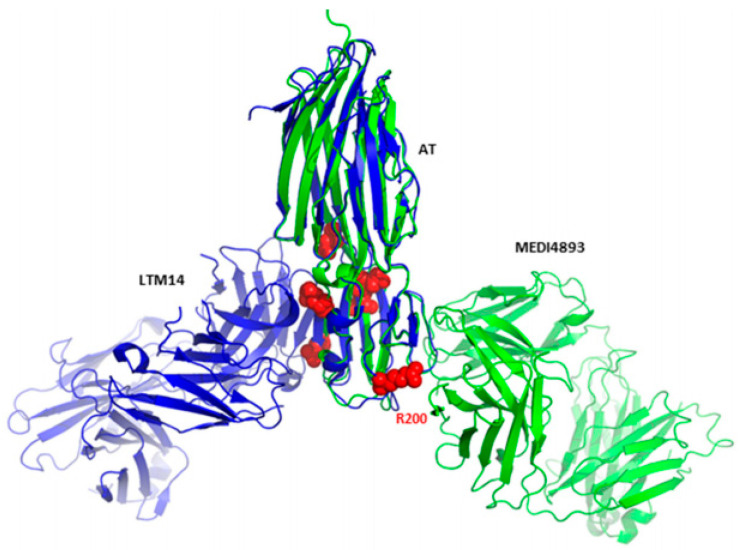
Superimposition of monomeric AT bound to MEDI4893 Fab (green) with monomeric AT bound to mAb LTM14 (PDB code 4IDJ, blue). Both Fab molecules bind to the same rim domain, though on opposite sides. Residues known to be critical for AT interaction with the cell surface receptor are shown as red spheres. Adapted from Oganesyan et al. [86].

Further, this study interpreted the dual mechanism of action of MEDI4893. In addition to its ability to inhibit AT binding to its receptor, MEDI4893 can also bind to the opposite side of the rim domain when compared with another AT mAb named LTM14 (Figure 12).

This reveals another plausible mechanism in which MEDI4893 prevents pore formation by preventing AT molecules from occupying neighbouring positions. In particular, the MEDI4893 light chain creates a steric hindrance with the neighbouring AT protomer in the rim region, and its heavy chain restricts two additional AT protomers from extending their stem. According to a previous study, which described the importance of residue Arg200 for AT binding to cell membranes and cell lysis [142], Oganesyan et al. supposed in response that Arg200 is the functional site blocked by MEDI4893. Later, these hypotheses were verified by introducing specific mutations [109]. According to the results, residues K266 and N188 critically contribute to MEDI4893 binding affinity to AT, and residue R200 was important for AT cell lysis, in agreement with the previous study [142].

### 3.4. Other Targets for Anti-Biofilm Treatment

In addition to the targets and their corresponding antibodies mentioned above, there are other types of anti-biofilm targets, including: (i) the cell wall-modifying enzymes and proteins, (ii) capsule and cell wall components and (iii) ATP-binding cassette (ABC) transporters. The first category includes the *S. aureus* autolysin (Atl) and immunodominant staphylococcal antigen A (IsaA), which are involved in cell division, growth and biofilm formation [143,144]. In the second category, lipoteichoic acid (LTA) and wall teichoic acid (WTA), as well as the capsular polysaccharides (CP) all contribute to the fundamental aspects of Gram-positive bacterial physiology [145,146]. As for the final category, the ABC transporter is responsible for cellular transport processes and drug resistance [147]. Studies on their antibodies are summarised in Table 1.

## 4. Conclusions and Future Outlook

This review aimed to illustrate the possibility of generating anti-biofilm antibodies for IE treatment, and a range of potential targets are mentioned and discussed above. According to the current studies, it can be summarised that biofilm formation is one of the major barriers that cause the persistence and drug resistance of IE. The *S. aureus* biofilm can form on both native and prosthetic valves. It has been shown that surface roughness is an important factor in stabilising cellular attachment to surfaces followed by biofilm formation [17]. In the case of NVE, bacterial infection causes vegetations on valve leaflets and the surrounding areas, which can increase the roughness of cardiac tissue and thus further promote the biofilm formation (see Section 1.2). Further, the most common material used to produce bioprosthetic valves is either bovine or pig pericardium, which are characterised by highly fibrous surfaces [104]. On the other hand, in comparison with antibiotic treatments, the major benefit of the antibody-based anti-biofilm strategy is the lower selection pressure it effects on bacteria, thus preventing further development of drug resistance. Moreover, it can both prevent the incidence of infection and directly intervene in the ongoing development of infections, and it can also be used as a supplementary therapy with antibiotics.

Amongst these candidates, two members of MSCRAMMs (ClfA and FnBP), two components of the biofilm matrix (PNAG and HU) and AT are mainly discussed. The first two candidates from the MSCRAMM family have been considered as the least promising for producing antibodies due to their poorly clarified shear stress-dependent binding mechanism. Furthermore, small differences in their composition can have large effects on antigenicity, shown as ClfA presenting lower binding affinities to antibodies elicited by other ClfA variants. As for the FnBPs, they even exhibit a much greater level of diversity in the A region subdomain to which their ligands Fg and Fn bind.

The three other targets, PNAG, HU and AT, are all considered promising candidates for generating antibodies due to their high accessibility of epitopes and conserved expression amongst different *Staphylococci* strains, which could also be combined in multimechanistic therapies (e.g., bispecific antibodies neutralising both HU and AT or bi-antibodies targeting both DNAP and dDNAP) [148]. Antibodies against *S. aureus* AT have been applied as a part of a combination therapy for IE in some previous studies, together with antibiotics and anti-ClfA antibodies [45,50,149]. AT is a reliable target for developing antibodies, with a robust body of evidence built since 1951. Furthermore, two anti-AT antibodies have already successfully completed a phase II clinical trial.

Taking all data together, targeting biofilms using antibodies can be considered an extremely promising treatment option for IE. Nonetheless, the development of natural antibodies is burdened by several limitations, such as short shelf-life, high costs of manufacturing and relatively low stability [150,151,152,153,154]. The ongoing development of nanotechnology tools for medical use might provide a suitable solution. Recently, the use of molecularly imprinted polymeric nanoparticles (MIP NPs) has been considered a reliable alternative strategy to conventional antibodies for their antibody-mimicking properties. Although this technology is still in its infancy for therapeutic applications, there is a growing body of evidence that indicates the potential feasibility of this approach [155,156,157,158,159,160,161,162,163,164].

## Figures and Tables

**Table 1:** Studies and clinical trials on potential anti-biofilm targets and antibodies. Adapted with permission from Raafat et al. [42]. 2019, Elsevier.

Antibody and Target				Clinical Trial			
Targets	Antibody Functions	Antibody	Refs	Name [Company; NCT Number]	Status	Intervention	Refs
ClfA	Block Fg binding/agglutination of human plasma; displace FBG-bound bacteria; promote OPK	Mu/mAb (mAb 12-9, 11H10); Huz/mAb (Tefibazumab)	[44,51]	Tefibazumab (Aurexis) [Inhibitex]	Phase II (failed)	Huz/mAb (IgG1)	[52]
				Tefibazumab (Aurexis1) [Inhibitex; NCT00198289]	Phase IIa (failed)	Huz/mAb (IgG1)	[53]
FnBPB	Block Fn binding; promote OPK and nGr activation; reduce biofilm formation	Mu/mAb	[54,55]				
Cna	Block CN binding; displace CN from bacterial surface; promote OPK; block laminin and C1q binding	Mu/pAb; Mu/mAb	[56,57,58]				
SasG	Reduce biofilm formation	Ra/pAb	[43]				
Atl	Inhibit biofilm formation; promote OPK	Mu/pAb	[59,60,61]				
Atl-Amd	Promote OPK	Mu/pAb	[60]				
Atl-Gmd	Promote OPK; block bacterial division (binary fission); induce agglutination	IgG1 Mu/mAb (1C11)	[62]				
IsaA	Promote nGr activation (oxidative burst) and OPK by nGr (UK-66); promote OPK in whole blood (hUK-66); promote nGr activation, but not phagocytosis (1D9)	Mu/mAb (UK-66); Huz/mAb (hUK-66); Hu/mAb (1D9)	[63,64,65,66]				
WTA	Promote C3 deposition and OPK by nGr (Hu/pAb)	Hu/mAb; IgG Hu/mAb (THIOMAB)	[67,68]	DSTA4637S [Roche/Genentech; NCT03162250]	Phase Ib (ongoing)	THIOMABTM antibody (Hu/mAb; IgG1)-antibiotic conjugate	[69,70]
CP	Promote OPK (Mu/mAb)	Mu/mAb; Ra/pAb; Mu/pAb	[71,72]	AltaStaphTM [Nabi Biopharmaceuticals; NCT00063089]	Phase II (halted)	Polyclonal human IgG	[73]
				AltaStaphTM [Nabi Biopharmaceuticals; NCT00066989]	Phase II (failed)	Polyclonal human IgG	[74]
LTA	Promote OPK	Murine/human chimeric mAb (Pagibaximab)	[75]	Pagibaximab1 [Biosynexus; NCT00631800]	Phase II (finished)	Murine/human chimeric mAb	[76]
				Pagibaximab1 [Biosynexus; NCT00646399]	Phase III (failed)	Murine/human chimeric mAb	[77]
PNAG/ dPNAG	Promote OPK	IgG1 Hu/mAb (F598)	[78,79]	SAR279356 [Sanofi-Aventis; NCT01389700]	Phase IIa (terminated due to difficulty in patient recruitment)	Hu/mAb	[80]
DNABII	Disrupt established biofilms	Native Hu/mAb (TRL1068)	[81,82]	TRL1068 [Trellis BioscienceLLC; NCT04763759]	Phase I (recruiting)	Hu/mAb	[83]
AT	Neutralise toxin activity; modestly inhibit biofilm formation	Hu/mAb (MEDI4893)	[84,85,86]	MEDI4893 (Suvratoxumab) [MedImmune LLC; NCT02296320]	Phase II (successful)	Hu/mAb (IgG1)	[87]
				AR-301 (Salvecin) [Aridis Pharmaceuticals; NCT01589185]	Phase IIa (successful)	Hu/mAb (IgG1)	[88]
LukAB	Neutralise LukAB-mediated cytotoxicity; inhibit LukAB binding to I domain of CD11b	Hu/mAb (SA-13, -15 and -17)	[89]				
GrfA	Reduce colonies in organ	Recombinant human scFv	[90]	Aurograb [NeuTec Pharma Ltd/Novartis Pharma AG; NCT00217841]	Phase II (failed)	Single-chain antibody fragment (Fab)	[57,90]
PhnD	Inhibit biofilm formation under shear flow (*S. aureus* and *S. epidermidis*), promote OPK by nGr	Ra/pAb	[91]				

## Data Availability

Not applicable.
